# Psilocybin-assisted psychotherapy as a potential treatment for eating disorders: a narrative review of preliminary evidence

**DOI:** 10.47626/2237-6089-2022-0597

**Published:** 2024-11-26

**Authors:** Elena Koning, Elisa Brietzke

**Affiliations:** 1 Queen's University Centre for Neurosciences Studies Kingston ON Canada Centre for Neurosciences Studies (CNS), Queen's University, Kingston, ON, Canada.; 2 Queen's University School of Medicine Department of Psychiatry Kingston ON Canada Department of Psychiatry, Queen's University School of Medicine, Kingston, ON, Canada.

**Keywords:** Psilocybin, eating disorders, psychedelics, psychotherapy, anorexia nervosa, eating behavior

## Abstract

Eating disorders (ED) are a group of potentially severe mental disorders characterized by abnormal energy balance, cognitive dysfunction, and emotional distress. Cognitive inflexibility is a major challenge to successful ED treatment and dysregulated serotonergic function has been implicated in this symptomatic dimension. Moreover, there are few effective treatment options and long-term remission of ED symptoms is difficult to achieve. There is emerging evidence for the use of psychedelic-assisted psychotherapy (PAP) for a range of mental disorders. Psilocybin is a serotonergic psychedelic that has demonstrated therapeutic benefit in a variety of psychiatric illnesses characterized by rigid thought patterns and treatment resistance. The current paper presents a narrative review of the hypothesis that psilocybin may be an effective adjunctive treatment for individuals with EDs, based on biological plausibility, transdiagnostic evidence, and preliminary results. Limitations of the PAP model and proposed future directions for its application to eating behavior are also discussed. Although the literature to date is not sufficient to propose the incorporation of psilocybin in the treatment of disordered eating behaviors, preliminary evidence supports the need for more rigorous clinical trials as an important avenue for future investigation.

## Introduction

Eating disorders (ED) are a group of potentially debilitating conditions in which the natural order of food intake, energy balance, and psychosocial functioning is disturbed.^1,2^ Their prevalence is rising, especially since the onset of the coronavirus disease 2019 (COVID-19) pandemic, and EDs have the highest mortality rate of any psychiatric disorder, estimated at between 10-15%.^3-6^ It is thought that complex genetic and environmental factors contribute to the pathology, including alterations to interoception and emotional-cognitive function.^7^

One aspect of ED pathology is dysregulation of serotonergic signaling, including in regions involved in appetite and reward, which has been associated with mood instability, body image distortion, and impulsivity in EDs.^8,9^ Additionally, altered functional connectivity in large intrinsic networks has been linked to abnormal executive function and cognitive inflexibility in this population.^9^ These neurobiological changes contribute to highly rigid beliefs and thought patterns in EDs, manifesting behaviorally as calorie counting, restrained emotional expression, and punishing exercise regimens.^10^ Further, other biological abnormalities have also been observed, such as low levels of brain-derived neurotrophic factor (BDNF), a neurotrophin involved in the regulation of energy homeostasis and food intake.^11^

There is currently little consensus on a first-line therapeutic model for EDs.^12^ Treatment typically includes psychosocial interventions and pharmacotherapy, in which the goal is to overcome dysfunctional beliefs and restore healthy eating behaviors and weight.^13,14^ However, long-term cessation of ED behaviors is difficult to achieve, considering the significant rates of avoidance, drop-out, and treatment resistance.^15-18^ As a result, many patients suffer a chronic course of disordered eating.^9^ Further, psychological factors pose a significant challenge, including cognitive inflexibility, altered interoception, and personality factors such as perfectionism, low self-compassion, and low existential well-being.^19,20^ Overall, there is a lack of evidence-based methods available to promote long-term therapeutic change in individuals with EDs.^15,16^

Alternatively, evidence is emerging for the use of psychedelic medicines in psychiatry, as an adjunct to psychotherapy.^21-23^ One survey indicated that 70% of people have tried complementary treatments to manage their EDs and the survey participants believed that psychedelic medicine is a valuable avenue for further investigation this group of disorders.^19^ Psychedelic medicines have been shown to promote positive shifts in body perception and reduced ED thoughts in preliminary and anecdotal reports.^24^ Psilocybin-assisted psychotherapy has been deemed safe and effective in treatment-resistant depression. Further, it may target mechanisms responsible for current treatment challenges in EDs, such as dysfunctional psychological rigidity. Previous research syntheses highlight the potential of psychedelic-assisted therapies for the treatment of EDs.^25,26^ However, there is yet to be a narrative review discussing the specific applications of psilocybin for eating behavior.

The objective of this narrative review is to present the hypothesis that psilocybin-assisted psychotherapy is a promising treatment for disordered eating behaviors, based on current evidence of the pathophysiological underpinnings of EDs, the mechanisms of action of psilocybin, and successful outcomes in other psychiatric disorders. In addition, this review will also outline preliminary evidence from investigations of psilocybin-assisted psychotherapy in EDs, address current criticisms and limitations of the field, and provide suggestions for future research initiatives.

## The biological plausibility of psilocybin-assisted psychotherapy

### Mechanism of action

Psychedelics have been used by humans for medicinal purposes for thousands of years. In particular, psilocybin, a naturally occurring plant alkaloid found in the *Psilocybe* genus of mushrooms, was introduced to western medicine by indigenous communities in the 1950s.^27,28^ More recently, clinical research has provided evidence of beneficial outcomes following psychedelic-assisted psychotherapy (PAP) in a range of mental disorders, including treatment-resistant depression, posttraumatic stress disorder, and substance use disorders.^29,30^ The PAP model includes a variety of modifications of standard psychotherapy including rigorous screening, preparation before each session, psychedelic dosing during the sessions, and an integration period afterwards. There are also unique contextual factors such as the influences of prior expectations or "set," as well as the environmental and social setting in which the therapy takes place.^31^

As a classical psychedelic, psilocybin is chemically similar to the neurotransmitter serotonin. Upon ingestion, it becomes dephosphorylated to the active metabolite psilocin that then crosses the blood-brain barrier and acts as an agonist of serotonin receptors, most notably 5-HT_2A_.^32^ The effects of psilocybin on the central nervous system are widespread and are thought to influence multiple neural networks and neurotransmitters, including restoration of serotonergic signaling in states of dysregulation.^33,34^ Psilocybin also alters cortical activity and increases connectivity between large intrinsic networks. Specifically, decreased activity in the default mode network (DMN), executive control network (ECN), and salience network has been linked to increased connectivity between these circuits, resulting in a brain state of higher cognitive flexibility.^33,35^ This is one way in which psilocybin acts as a treatment for mental illnesses involving highly constrained or rigid thought patterns.^21^

**Figure 1:**
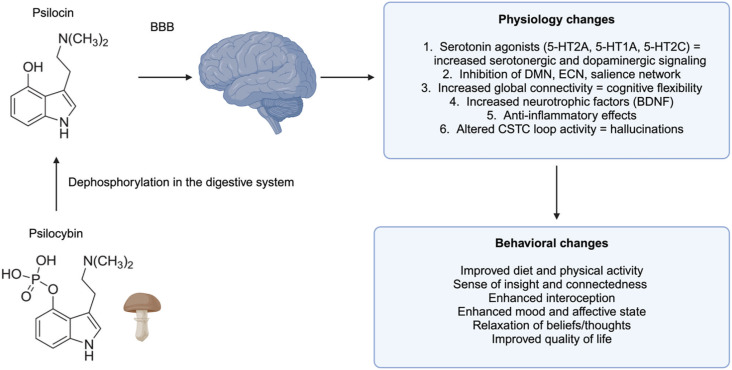
The mechanism of action of psilocybin and related physiological effects in the central nervous system, producing potential therapeutic behavioral changes. Figure created with Biorender.com. BBB = blood-brain barrier; BDNF = brain-derived neurotrophic factor; DMN = default mode network; CSTC = corticostriatal-thalamo-cortical; ECN = executive control network.

These neurophysiological changes have been linked to broad and favorable psychological results. Specifically, psilocybin promotes openness, relaxation of prior beliefs, and improved interoception.^21^ In 2019, Carhart-Harris and Friston^36^ introduced the RElaxed Beliefs Under pSychedelics (REBUS) model to describe the effects of classical psychedelics as a "loosening" or "lubricating" of rigid thinking and prior expectations which is hypothesized to contribute to a state of improved bottom-up information flow. In EDs, this could be conceptualized as a restoration of healthy thought patterns in relation to eating behavior and body image. Increased levels of BDNF are also implicated in the sustained behavioral benefits of psilocybin administration.^36^ Activation of 5-HT_2A_ receptors via psilocybin is also known to have anti-inflammatory effects.^21^ Meanwhile, altered activity in the corticostriatal-thalamo-cortical (CSTC) loop underlies the hallucinatory properties of this substance.^37^ Figure 1 presents a schematic illustrating the mechanisms of action of psilocybin and potential behavioral changes.

Psilocybin and other serotonergic psychedelics have a relatively low risk profile. Although persistent psychological complications have been reported, adverse events are rare, especially with proper screening.^38-40^ Minor, acute physiological changes may include increased heart rate and blood pressure, as well as nausea and headache.^41^ Transient anxiety is a common psychological side effect, although this can be properly managed through dosage, ongoing psychological support, and contextual considerations.^42^ Often, challenging experiences under the influence of psychedelics are associated with positive learning opportunities and have been linked to the therapeutic benefit of their use in the psychotherapeutic setting.^40,43,44^

### Psilocybin and improvements in eating behavior

Serotonin receptors are widely expressed in brain regions that regulate hunger, satiety, eating rate, impulsivity, inhibition, attention, and mood. Psilocybin has been shown to enhance synaptic plasticity, which may overcome rigid prior belief and behavioral patterns in individuals with EDs. Serotonergic activation via psychedelics has been shown to promote long-term behavioral changes in lifestyle, including eating behaviors (Figure 1). For example, psilocybin use is associated with reduced body mass index (BMI), increased consumption of fruits and vegetables, and increased levels of physical activity.^35^ In a recent study, a single dose of psilocybin was shown to reduce sucrose preference in mouse models of obesity.^45^ Although no effects on food intake were observed, the authors highlighted a need for additional studies in humans. Case studies have reported positive changes in personality and affective state following psilocybin administration, including an increased sense of connectedness, openness, and introspection. Enhanced mood state and quality of life are also often routinely reported.^22^ Therefore, psilocybin-assisted psychotherapy might contribute to improved eating behavior outcomes and psychological well-being in individuals with EDs.

## Current uses for psilocybin in psychiatry

The field of psychiatry has adopted a largely taxonomic approach to diagnosis and treatment, as outlined in the Diagnostic and Statistical Manual of Mental Disorders, 5th edition (DSM-5). However, it is not uncommon for medications and treatments originally developed for one condition to be repurposed to treat others. The transdiagnostic approach introduces a new form of classification based on common etiological, behavioral, and cognitive-affective aspects of mental illnesses and is relevant to the use of psychedelics in psychiatry.^46,47^ Psilocybin has already been studied in a variety of psychiatric contexts and yields therapeutic benefit in illnesses that demonstrate overlap in the proposed pathophysiological mechanisms associated with EDs.

One of the most well-studied uses for psilocybin in psychiatry is as an adjunctive treatment for depression. The beneficial effects of psilocybin-assisted psychotherapy on mood include destabilization of constrained, negative thought patterns, as well as enhanced mood stability and reduced suicidal ideation.^37,48,49^ Significant improvements in both depressive and anxiety symptoms occurred following two sessions of psilocybin administration with sustained therapeutic effects at 3-month follow-up.^34^ Furthermore, in 2018, the Federal Drug Administration (FDA) identified psilocybin as a breakthrough therapy for treatment-resistant depression, and several clinical trials have been conducted to date around the world.^28,50,51^ The mood enhancing effects of psilocybin can be attributed to altered activity in large intrinsic networks such as the DMN, in addition to the amygdala and ACC, in which altered neural activity is correlated with reduced depressive symptoms.^36,52^ Research has also linked these results to enhanced emotional control following psilocybin administration.^53^

Individuals with cancer or other terminal illnesses have also demonstrated beneficial outcomes from psilocybin-assisted psychotherapy, most notably reduced end-of-life anxiety, mood symptoms, and fear of death.^22,37,50,54,55^ Specifically, in one study, psilocybin use by cancer patients with comorbid depression and generalized anxiety disorder resulted in clinical response rates of approximately 80% at 6-month follow-up.^49^ Improvements in hopelessness, demoralization, and paranoia were also reported. Enhanced feelings of relief, interconnectedness, and self-perception are implicated in the beneficial effects of psilocybin on death-associated anxiety and depression, particularly due to the mystical and spiritual aspects of the experience.^22,55,56^

Psilocybin treatment has also been studied as an adjunctive treatment for individuals with addiction and substance-use disorders (SUD).^34,36,50,57^ For example, a recent study of psychedelic use in SUD resulted in reduced craving behavior in all participants.^22^ Another study demonstrated that 80% of individuals with nicotine addiction were abstinent at 6-months following three sessions of psilocybin administration.^33^ Similar results have also been observed in alcohol addiction.^36,37^ The anti-addictive effects of psilocybin have been linked to altered dopaminergic and serotonergic signaling, including in reward circuits.^36^

Finally, there is potential use for psilocybin in post-traumatic stress disorder (PTSD). Although the pathophysiology of PTSD is not completely understood, abnormal neural connectivity is characteristic of this disorder as well as altered limbic activity and neurotransmitter actions.^58^ Psilocybin has been linked to increased emotional empathy, cognitive flexibility, and memory consolidation, which is of benefit to individuals suffering from PTSD as patients with emotional detachment are less likely to improve. Both human studies and animal models of PTSD have demonstrated therapeutic benefit from psilocybin, including enhanced mood and reduced threat-associated neural activity.^58,59^

## Preliminary evidence and ongoing research in eating disorders

The use of psychedelics in psychiatry is widespread and growing. In application to EDs, there is a growing body of clinical research on PAP conducted to date. For example, intermittent ketamine infusions successfully treated compulsive behavior in EDs.^60^ More recently, a cohort study indicated that individuals with a lifetime prevalence of EDs had profound reductions in depressive scores and increased well-being after psychedelic administration.^61^ Additionally, a qualitative study showed reduced or complete cessation of ED symptoms and negative thoughts following ayahuasca.^24,62^ Improvements in neurocognitive functioning, mood, and self-love, as well as reduced impulsivity have also been linked to psychedelic use.^33,62^ Psilocybin is often considered the psychedelic of choice for adjunctive psychiatric treatment due to its designation as safe and effective for the treatment of depression.^9^ Although limited clinical research has applied psilocybin in PAP for EDs, existing preliminary and anecdotal findings indicate positive results. Theoretical mechanisms of action of psilocybin in EDs suggest the potential to relieve symptoms of altered serotonergic signaling and cognitive inflexibility, acting as a catalyst to the often-challenging psychotherapeutic process in this patient population.

### Psilocybin and anorexia nervosa

Although the pathophysiological basis of AN remains elusive, disturbed neurotransmitter signaling may form part of its etiology. Specifically, serotonergic networks predict the onset of AN and may contribute to altered eating behavior and mood.^63^ In addition, neuroimaging studies have revealed neurostructural alterations in AN, as well as abnormalities in reward and somatosensory processing.^64,65^ It is hypothesized that psilocybin might improve serotonergic function, maladaptive eating behaviors, and depressive symptoms in AN.^61^ In addition, psilocybin-assisted psychotherapy has been linked to enhanced cognitive flexibility, which could potentially reduce suicidality and treatment resistance in this patient group. Furthermore, improvements in anxiety and mood following psilocybin administration have been linked to reduced avoidance of food-related stimuli and fear of weight gain in AN, promoting long-term recovery.^61^

Psilocybin has already demonstrated benefit in animal models of AN, by restoring serotonergic function. Psilocybin has also been successful in the treatment of AN comorbidity, including anxiety, depression, SUDs, and PTSD. Suicidality is a main cause of mortality in AN and MDD is comorbid in 50-75% of cases.^9^ Therefore, the established antidepressant effects of psilocybin-assisted psychotherapy may be valuable for ED cases with a comorbid mood disorder. Further, traumatic memories are known to contribute to increased symptom severity and fear of weight gain in AN, while psilocybin administration reduces neural activity in regions involved in processing fear and threatening stimuli.^9^ Psilocybin has also been shown to treat experiential and emotional avoidance aspects in AN.

A 1959 French case study described a woman with treatment-resistant AN who received two doses of psilocybin, causing increased perceived insight into the root cause of her symptoms, immediately increasing mood, with long-term weight resolution.^66^ More recently, three ongoing clinical trials are evaluating psilocybin-assisted psychotherapy in individuals with AN. In collaboration with COMPASS Pathways, the University of California is conducting a Phase 2 interventional trial in which 16 participants will receive 25 mg of oral psilocybin with psychological support.^67^ In addition, COMPASS Pathways is actively recruiting 60 participants for a Phase 2, randomized, parallel assignment trial comparing psilocybin-assisted therapy at 1 mg and 25 mg doses.^67,68^ Another open-label interventional trial is currently underway at Imperial College London, in which 20 participants will receive three doses of up to 25 mg of psilocybin along with psychological support. This study will also examine the neuronal underpinnings of psilocybin treatment through magnetic resonance imaging (MRI) and electroencephalography (EEG) approaches.^69^ Finally, Johns Hopkins University is conducting a Phase 1 open-label clinical trial using experimental psilocybin (up to four doses) in 18 participants with AN.^70^

### Psilocybin and bulimia nervosa

Bulimia nervosa (BN) is also characterized by binge eating in addition to compulsive behaviors in order to compensate for overeating such as laxative use, starvation, and self-induced vomiting.^71^ With prevalence rates of up to 3% in females, BN is often described as a chronic phase of AN and is thought to have even worse prognosis, while also being more resistant to treatment.^72^ A resting-state functional neuroimaging study indicated abnormal global and network-level connectivity in women with BN, including sensorimotor, visual, and limbic systems, which may explain pathological eating behaviors and body image.^73^ It is possible psilocybin may improve symptoms of BN by improving cognitive rigidity and network connectivity, which contribute to unhealthy thought patterns and compensatory behavior in these patients. Psilocybin-assisted reduction of impulsive overeating behaviors may improve BN symptomology. However, no clinical studies have been conducted to date and, therefore, there is insufficient evidence to support a therapeutic effect of psilocybin for individuals with BN.

### Psilocybin and binge eating disorder

Binge eating is another form of compulsive eating behavior with similar mechanisms as previously described. Binge eating disorder (BED) is characterized by recurrent episodes of overeating and a sense of loss of control, including the consumption of objectively large amounts of food in a short period of time. There is evidence to suggest that enhanced food reward in concert with increased impulsivity may constitute the neurobiological basis of BED.^74^ Patients demonstrate altered serotonergic signaling in a variety of brain regions and selective serotonin reuptake inhibitors (SSRIs) have already been considered for their potential therapeutic actions in this patient group.

It has been suggested that BED-associated rumination and intrusive thoughts may benefit from the use of psychedelic medicine.^75^ In addition, psilocybin improves impulsivity and mood state, which may be therapeutic to individuals with BED by limiting emotional eating, a key contributor to bingeing.^76^ As a 5-HT agonist, psilocybin may also exhibit therapeutic benefit to abnormal neurotransmitter signaling in BED, although this has yet to be experimentally validated. The safety and feasibility of a study evaluating psilocybin-assisted psychotherapy is being investigated in a Phase 2 open-label clinical trial sponsored by TRYP Therapeutics. In this study, 10 participants with BED will receive a 25 mg dose of psilocybin along with psychological support and will be followed up 5 months post-screening.^77^

### Psilocybin and food addiction

Food addiction is an emerging concept in psychiatry, which is estimated to impact about 16-20% of adults.^78^ It consists of behaviors that closely exhibit those of SUDs, including excessive craving, enhanced food reward, and impulsive food consumption. Food addiction demonstrates overlap with obsessive-compulsive eating (OCE) behaviors and may contribute to development of obesity and metabolic syndrome and to significant reductions in quality of life.^79^ Interestingly, the same neural circuits underlie substance abuse and food addiction and psilocybin may therefore have potential as a treatment for addictive eating behaviors through its actions on the mesolimbic system.^35^ The therapeutic actions of psilocybin have already been demonstrated in application to drug addiction and may be linked to the mystical aspects of the experience. In fact, higher mystical-type experience scores have been shown to correlate with a greater reduction in craving behavior.^34^

Another proposed mechanism for the benefit of psilocybin in food addiction is related to altered activity in the ECN. Specifically, cortisol levels increase following psilocybin treatment, activating this brain network and increasing control over emotional processes including relief of persistent negative thinking.^80,81^ Microdosing psilocybin has demonstrated spontaneous improvements in exercise and eating habits in addition to reduced addictive behavior.^35^ One study has suggested a role for psychedelics in food addiction,^33^ although no clinical studies have been conducted to date and there is limited evidence available to support their use.

### Psilocybin and body image

Body image issues are a key characteristic of EDs, a domain of psychopathology shared with body dysmorphic disorder (BDD), a mental disorder characterized by an imagined defect in appearance and obsession with one's body image, causing emotional distress, suicidality, and functional impairments.^82^

The somewhat successful treatment of BDD with SSRIs supports the hypothesis that dysregulated serotonin signaling forms part of the etiological basis of the disorder. Therefore, the serotonergic actions of psilocybin indicate potential for this substance in BDD treatment. One case study described an individual with BDD who responded well to both the SSRI fluoxetine and psilocybin treatment, but was treatment-resistant to a variety of other medications.^83^ The patient reported cessation of their perceived body distortions following psilocybin therapy. Both psilocybin and fluoxetine have high affinity for the serotonin system, indicating a potential overlap in the therapeutic actions of these medicines. The mystical effects of psilocybin have been linked to increased sense and acceptance of self, which may improve distorted self-image and body perception in BDD. A Phase 2 open-label pilot study is currently undergoing recruitment in which a single dose of psilocybin along with psychological support will be administered to individuals with BDD who have not had success with at least one trial using SSRIs.

### Psilocybin and obsessive-compulsive eating

Obsessive-compulsive behavior lies on a spectrum of disorders sharing features with obsessive-compulsive disorder (OCD), including obsessive thoughts, compulsive behavior, and anxiety. Both human and animal studies of OCD have demonstrated therapeutic benefit from psilocybin.^21,36,50^ In humans, multiple small-scale studies have demonstrated statistically significant effects of psilocybin on OCD-related symptom reduction, linked to its actions on serotonergic signaling and cerebral connectivity.^37,76^ In EDs, multiple pathophysiological mechanisms are shared with OCD. OCE is defined as an unhealthy fixation with food, as well as compulsive eating behaviors, such as habitual or emotional overeating.^84^ OCE behaviors are considered an underlying contributor to obesity and metabolic disorders. One proposed mechanism of OCE behaviors is a loss of inhibitory control mechanisms through dysregulated neurotransmitter and neuropeptide signaling, including serotonergic.^85^

Psilocybin has already been shown to improve comorbid obsessive eating dysfunction in mood disorders.^33,86^ In addition, a recent study described a potential role for psilocybin in the treatment of compulsive overeating and obesity.^21^ Dysfunctional serotonin and dopamine signaling is characteristic of obsessive-compulsive behavior, including abnormal activity in the CSTC circuit, which plays a role in fear, obsessions, unattainable rules, and compulsions. The DMN also mediates deliberate negative thoughts and is linked to an inability to focus attention on external cues. The anti-obsessional effects of psilocybin have been shown to act immediately on the aforementioned mechanisms, disrupting the CSTC circuit and prompting neuroplasticity in the DMN, in addition to other beneficial actions.^75^

**Figure 2:**
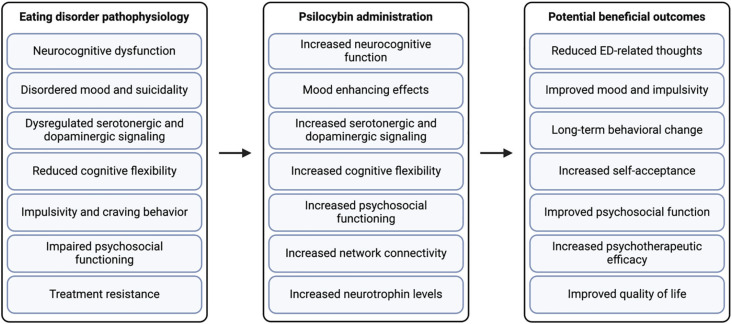
The potential therapeutic actions of psilocybin in eating disorders. Figure created with Biorender.com. ED = eating disorder.

Figure 2 depicts a summary of the potential therapeutic actions of psilocybin-assisted psychotherapy on eating behavior outcomes.

## Limitations of preliminary evidence and future directions

Although there is promising evidence emerging for the use of psilocybin-assisted psychotherapy in psychiatry, it is important to consider the limitations of the evidence accumulated thus far. First, current studies are largely case-based, anecdotal, or theoretical in nature. There is a lack of large-scale randomized clinical trials applying psilocybin to psychotherapy for EDs. Moving forward, methodological issues associated with PAP should be considered, including the lack of long-term follow-up and the characteristics of samples, which are typically small and lacking in diversity.^87^ Further, the subjective perceptual effects of the psychedelic experience pose a significant challenge to blinding in these studies. Some studies may attempt to mitigate this limitation by including an inert placebo condition such as low dose psilocybin (e.g., 1 mg). In addition, the comparison of psilocybin with other psychedelic substances may help discern therapeutic benefit, such as comparison to infusion of ketamine, a dissociative anesthetic, which is already approved as an adjunct to psychotherapy.^88^ Another limitation is the potential influence of researcher bias or "excessive enthusiasm," in which the personal use of psychedelics by researchers may compromise scientific objectivity and promote biased reporting of results.^89^

It is important to gain a clear understanding of the underlying mechanisms of psilocybin to truly identify its safety and therapeutic actions in EDs. Although current knowledge is promising, it is often difficult to discern which aspects of the psilocybin experience are contributing to beneficial outcomes. For example, shifted cognition, enhanced mood, altered network connectivity, psychological flexibility, serotonergic actions, and mystical-type experiences are all influential actions of psilocybin and may play independent or convergent roles in the treatment of EDs.^55^ Furthermore, the subjective and context-dependent nature of the psychedelic experience adds to this complexity. Specifically, the use of psilocybin in ED treatment may produce different results depending on the patient, environment, and social nature of substance administration. For example, every patient demonstrates a unique set of traumas, severity of ED symptoms, and comorbidities.

The approach to administering proper informed consent is another ethical challenge due to the ineffable nature of the psychedelic experience, which often makes it difficult to portray to naïve users.^90^ In addition, the relationship between patient and psychotherapist is one of the strongest predictors of therapeutic outcome and the effects of this alliance on psilocybin efficacy warrants further investigation.^33^ Inappropriate use of therapist power, including sexual misconduct, is another concern among psychedelic clinical trials, highlighting the importance of developing specific guidelines and regulatory frameworks for practitioners.^91^ Finally, contextual factors such as set and setting during dosing, as well as preparatory and integration components of the PAP model, should consider a personalized approach. This will aid in reducing unwanted influences and the risk of adverse psychological events during dosing.

As it stands, we are on our way to uncovering the therapeutic actions of psilocybin-assisted psychotherapy and its potential application to EDs. Future research should focus on identifying the precise therapeutic mechanisms of psilocybin on eating behavior, body image, and mood, as well as long-term efficacy in ED populations. As a substance with widespread effects, the transdiagnostic use of psilocybin may be beneficial to ED patients with comorbid conditions. For example, mood disorders and EDs have a high rate of comorbidity and psilocybin-assisted psychotherapy is demonstrating positive outcome for individuals with treatment-resistant depression. Further, maladaptive eating behaviors are widespread in mood disorders and contribute to illness severity even when below the threshold for diagnosis of an ED.^92^ It is recommended that future clinical trials investigate eating behavior outcomes in non-ED psychiatric patient samples as well. This may aid in avoiding the implementation of multiple pharmacotherapies, which often carry numerous adverse side effects each and do not usually address the core pathophysiology of EDs.

In addition to conducting more randomized controlled trials, the results of observational or naturalistic studies are also important to consider as supporting evidence. There is also much to learn from the ceremonial use of psilocybin and it is essential to involve indigenous peoples in the political and educational decisions regarding their use. Representation among both psychedelic researchers and participants is essential to ethical and proper generalization of results. Overall, there are many items to keep in mind before widespread implementation of PAP, including psilocybin-assisted psychotherapy in EDs.

## Conclusion

In conclusion, there is emerging evidence for the application of psilocybin-assisted psychotherapy to EDs. The literature to date indicates that psilocybin may be beneficial to mental disorders characterized by repetitive negative thinking and an inability to inhibit unwanted behaviors. These aspects are highly relevant to EDs and are reflected in the scarce preliminary evidence of therapeutic benefit. Psilocybin has demonstrated reduced ED-associated thoughts and an increased sense of direction for afflicted individuals, although most studies have only been conducted in AN. Despite the desperate need for alternative interventions for EDs, the field of psychedelic medicine is still early and emerging. There are a variety of knowledge gaps and limitations to be addressed through more rigorous clinical trial design, as well as proper screening and representation among both researchers and participants. Nonetheless, there is potential for this novel therapeutic strategy to significantly improve therapeutic outcomes for individuals with EDs and it can be considered an important avenue for future investigation.
